# Relationship between 24-h Ambulatory Blood Pressure Variability and Degree of Renal Artery Stenosis in Hospitalized Patients with Hypertension

**DOI:** 10.31083/j.rcm2511397

**Published:** 2024-11-08

**Authors:** Xiaoyang Luo, Wei Liu, Xi Peng, Pengqiang Li

**Affiliations:** ^1^Department of Cardiovascular, Beijing Hospital, National Center of Gerontology; Institute of Geriatric Medicine, Chinese Academy of Medical Science, 100010 Beijing, China; ^2^Department of Cardiovascular, The Second Hospital of Shanxi Medical University, 030000 Taiyuan, Shanxi, China; ^3^Department of Cardiovascular, Cardiac Arrhythmia Center, Chinese Academy of Medical Sciences, Peking Union Medical College, National Center for Cardiovascular Diseases, Fu Wai Hospital, 100010 Beijing, China; ^4^Department of cardiovascular, Peking University Third Hospital, 100010 Beijing, China

**Keywords:** hypertension, blood pressure variability, atherosclerosis, renal artery stenosis

## Abstract

**Background:**

Blood pressure variability (BPV) is a critical risk factor for cardiovascular outcomes and is associated with atherosclerotic renal artery stenosis (ARAS), which is diagnosed using digital subtraction angiography (DSA). However, the relationship between the degree of renal artery stenosis (d-RAS), diagnosed using renal artery contrast-enhanced ultrasound (CEUS), and 24-hour ambulatory BPV in hospitalized patients with ARAS remains unclear.

**Methods:**

Hospitalized hypertensive patients were divided into ARAS and non-ARAS groups based RAS diagnoses using CEUS. The ARAS patients were further classified into unilateral and bilateral categories. Quantification of BPV over 24 hours, daytime, and nighttime utilized standard deviation (SD), coefficient of variation (CV), and average real variability (ARV). Percentage stenosis was used to evaluate d-RAS. Pearson’s and multivariate beta regression analyses were used to assess correlations between BPV and d-RAS.

**Results:**

We found that 24-hour systolic BPV (SBPV), presented as SD, CV, and ARV indices, was positively correlated with unilateral d-RAS (R^1^ = 0.460, *p* = 0.001; R^1^ = 0.509, *p* < 0.001; R^1^ = 0.677, *p* < 0.001, respectively). This correlation was consistent with the daytime SBPV (R^1^ = 0.512, *p* < 0.001; R^1^ = 0.539, *p* < 0.001; R^1^ = 0.678, *p* < 0.001, respectively) and daytime diastolic BPV (DBPV) (R^1^ = 0.379, *p* = 0.010; R^1^ = 0.397, *p* = 0.007; R^1^ = 0.319, *p* = 0.033, respectively). Similarly, 24-hour DBPV assessed by SD and CV also correlated positively with unilateral d-RAS (R^1^ = 0.347, *p* = 0.019; R^1^ = 0.340, *p* = 0.022, respectively), as did nighttime SBPV assessed by ARV indices (R^1^ = 0.415, *p* = 0.005). No significant correlations were found between BPV and bilateral d-RAS (*p* > 0.05). Multivariate beta regression analysis indicated that 24-hour SBPV (odds ratio [OR] = 1.035, 95% confidence interval [CI]: 1.054–1.607, *p* = 0.035) and daytime SBPV (OR = 1.033, 95% CI: 1.004–1.061, *p* = 0.023; both evaluated via AVR) were independent risk factors for d-RAS.

**Conclusions:**

SBPV is positively correlated with unilateral d-RAS at all time points. Both 24-hour and daytime SBPV (evaluated using ARV indices) were identified as independent d-RAS risk factors.

## 1. Introduction

Hypertension is a known risk factor for atherosclerosis [1]. Traditional blood pressure (BP) management depends on serial office-based BP measurements or intermittent home monitoring. Despite effective BP control, some hypertensive patients still develop systemic arterial atherosclerosis, potentially linked to BP variability (BPV) [2]. Short-term BPV describes the normal physiological fluctuations in healthy adults, includes diurnal or short-term variations [2]. These can be quantified over 24 hours using various methods including invasive monitoring, office BP monitoring, ambulatory BP monitoring (ABPM), or home BP monitoring (HBPM) [2]. A recent study highlighted the risks associated with extreme BPV measured during the first day in intensive care unit (ICU), showing a higher mortality rate [3]. Further, Xianglin Chi *et al*. [4] reported a correlation between within-visit systolic BPV over 24 hours and internal carotid plaque in patients with hypertension who were undergoing antihypertensive therapy. ABPM is recommended as the most effective noninvasive method for assessing 24-hour BP fluctuations [5], and has been independently validated as a predictor of cardiovascular events [6]. Notably, Kawai *et al*. [7] found significant correlations between daytime and nighttime short-term BPV measured by ABPM and renal function, suggesting its utility in evaluating a broader spectrum of non-cardiovascular pathologies. Supporting this, the CMERC-HI study demonstrated that short-term BPV evaluated using ABPM is associated with development of composite kidney disease outcome in patients with hypertension [8]. Generally, we use three indicators to evaluate BPV, including standard deviation (SD), coefficient of variation (CV), and average real variability (ARV), but it is not clear which one is more reliable.

Atherosclerotic renal artery stenosis (ARAS) is a common cause of renal artery stenosis (RAS) and secondary hypertension, often leading to adverse cardiovascular outcomes and ischemic nephropathy [1]. While digital subtraction angiography (DSA) remains the gold standard for diagnosing RAS, its applicability is limited due to its high radiation dose, invasiveness, potential for allergic reactions to contrast agents, and nephrotoxicity [9]. In contrast, renal artery contrast-enhanced ultrasound (CEUS) is a recently developed diagnostic alternative for assessing RAS: it produces less trauma, avoids allergenicity, and has a minimal impact on renal function [10]. Previous studies have shown that CEUS has a sensitivity and specificity of approximately 90% compared with DSA and is an effective diagnostic method for RAS [11, 12]. Notably, patients with ARAS exhibit increased BPV and poorer prognoses [7]. However, the relationship between the degree of RAS (d-RAS) and BPV in ARAS patients diagnosed using CEUS remains underexplored. Therefore, this study aimed to investigate the characteristics of short-term BPV in patients with ARAS, examining its association with d-RAS, and identifying other influencing factors.

## 2. Methods

### 2.1 Study Population

The study recruited 122 patients aged 31–87 years with hypertension who were hospitalized at Beijing Hospital between January 2018 and December 2022. All conditions were in accord with the latest version of the Declaration of Helsinki [13]. The Institutional Ethics Committee of the Beijing Hospital approved this study (no. 2023BJYYEC-275-02). The inclusion criteria were as follows: (1) clinically diagnosed hypertension; (2) ABPM and renal artery CEUS results; and (3) >70% effective BP measurement within 24-hours. The exclusion criteria were: (1) primary hyperaldosteronism and obstructive sleep apnea syndrome; (2) inability to tolerate 24-hour ABPM or renal artery CEUS; (3) surgical treatment history for patients with ARAS; (4) daytime and nighttime reversal; and (5) severe heart failure, arrhythmia and valvular heart disease. All patients were divided into the ARAS group (62 patients) and the non-ARAS group (58 patients). Furthermore, ARAS patients were subdivided into unilateral (45 patients) and bilateral (19 patients) groups according to the location of renal artery stenosis.

### 2.2 Measurement and Definition

Residents obtained the medical history and performed physical examinations on all participants. The participants fasted for 8 h, and their elbow venous blood was withdrawn the next morning. The same automatic biochemical analyzer (Hitachi 7600, Hitachi high-tech corporation, Tokyo, Japan) was used to measure triglyceride (TG), low-density lipoprotein cholesterol (LDL-C), glucose (GLU), glycosylated hemoglobin A1c (HbA1c), creatinine (Cr), estimated glomerular filtration rate (eGFR), plasma renin activity (PRA), plasma aldosterone concentration (PAC), and plasma aldosterone-to-renin ratio (ARR) levels. The eGFR was calculated using the chronic kidney disease-epidemiology collaboration (CKD-EPI) formula (eGFR = 141 × min [serum creatinine (Scr)/κ,1] α × max [Scr/κ,1] – 1.209 × 0.993 age × 1.018 [for women] × 1.159). PRA and PAC were measured using an enzyme-linked immunoassay, the kits were purchased from Beijing North Institute of Biotechnology Company Co. Ltd. (Beijing, China) and operated according to the instructions. Participants who smoked >400 cigarettes in their lifetime were defined as having a smoking history [14]. People who drank three or more drinks per day were defined as having a drinking history [15]. Diabetes was diagnosed as glycosylated hemoglobin A1c ≥6.5%, previous diagnosis of diabetes, and/or chronic hypoglycemic drug-usage [16]. Coronary lumen diameter stenosis ≥50% in the main artery or its major branches confirmed by angiography, computed tomography angiography, or magnetic resonance imaging was diagnosed as coronary heart disease (CHD) [17]. BP at night is 10 to 20% lower than BP in the daytime, called dipper BP. A 0 to 10% decrease in BP at night compared to BP in the daytime is called non-dipper BP. The BP at night is less than 0% lower than the BP in the daytime, which is called reverse dipper BP. The reduction of BP at night by more than 20% compared to BP in the daytime is called super dipper BP [18, 19].

### 2.3 ABPM

Notably, all participants used the same type of ABPM (Vaso Medical CB-1805-B, Vaso Medical Company, Westbury, NY, USA) and selected a suitable cuff for ABPM on the non-dominant arm. The ABPM device had been validated independently according to the internationally accepted validation protocols. The device was worn for at least 24 h. The BP was measured automatically every 30 min during the daytime, from 8:00 to 21:59. Nighttime BP monitoring was performed hourly between 22:00 and 7:59. The effective reading should be more than 70% of the set reading, at least 20 active readings during the daytime and 7 at night [20]. Based on the recorded 24-hour BP measurements, BPV was evaluated by calculating the SD, CV, and ARV of systolic BP (SBP) and diastolic BP (DBP) during the daytime, nighttime, and over 24-hours. For the short-term (within 24-hours) BPV analysis, SD, CV, and ARV were the common BPV indices in the time domain.

### 2.4 Renal Artery CEUS

After the patients fasted for 8 h, their renal artery was evaluated using ordinary ultrasound (Samsung RS80A, Samsung company, Seongnam, Gyeonggi Province, Korea), and a peripheral intravenous ultrasound contrast agent was injected four times, 0.8–1.5 mL each time. After each injection, 5 mL of normal saline was used for irrigation, and the timing was initiated. Approximately 10–15 s later, blood perfusion in the main renal artery and kidney was observed during the cortical enhancement period. In the patient’s left decubitus position, a transverse section was taken from the right anterior abdominal intercostal space, and a coronal section was taken from the waist in the lateral decubitus position. The value was obtained at the point with the narrowest jet beam and fastest flow rate. Finally, a comprehensive diagnosis was made based on the conventional ultrasound and CEUS results. The patients could be diagnosed as ARAS, when they had at least one risk factor for atherosclerosis [21] (obesity, diabetes, hyperlipidemia, age >40 years, long-term smoking) and at least two radiographic findings of atherosclerosis [22] (plaque-like irregularities of the renal artery, tapered stenosis or occlusion, eccentric stenosis, calcification, mainly involving of the orifice or proximal portion of the renal artery; manifestations of atherosclerosis in other abdominal vessels). The d-RAS was expressed by the diameter stenosis rate, calculated as follows: diameter stenosis rate = (1 – contrast media width at the stenosis/contrast media width near the normal segment) ×100%.

### 2.5 Statistical Methods

IBM SPSS version 26.0 (IBM Corporation, Armonk, NY, USA) was used to organize and analyze the data. Patients were divided into ARAS and non-ARAS groups, and the clinical characteristics of the two groups were compared. Categorical variables were tested using the chi-square test and expressed as composition ratios. If the continuous variables were normally distributed and the variance was neat, the *t*-test was used, and values were expressed as the mean ± standard deviation. Conversely, a non-parametric test was adopted, and values were expressed as the median (25% and 75%). Pearson’s correlation analysis was used to analyze the correlation between the d-RAS and BPV. The closer the correlation coefficient R is to 1, the greater the correlation between the two variables. Univariate and multivariate Beta regression models were established using the R version 4.3.0 (The R Foundation for Statistical Computing, Vienna, Austria) to analyze the risk factors for RAS. Test level α = 0.05, and statistical significance was set at bilateral *p* < 0.05.

##  3. Results

This study evaluated 122 participants, with an average age of 67.62 ± 12.90 years and an average duration of hypertension of 18.45 ± 13.31 years, comprising 49.2% males. The cohort was divided into ARAS and non-ARAS groups included 64 and 58 patients, respectively. As shown in Table 1, the ARAS group not only included a higher proportion of older men, but also displayed longer durations of hypertension compared to the non-ARAS group. Additionally, The ARAS group exhibited significantly higher body mass index (BMI), Cr, and aldosterone-to-renin ratio (ARR) levels (*p* < 0.05). The eGFR levels were significantly lower in the ARAS group than in the non-ARAS group (*p* < 0.05). Notably, the 24-hour, daytime, and nighttime means SBP were elevated in the ARAS group (*p* < 0.05), a similar trend was observed in 24-hour, daytime, and nighttime systolic blood pressure variability (SBPV) and diastolic blood pressure variability (DBPV) (*p* < 0.001) (**Supplementary Table 1**).

**Table 1.** **Baseline characteristics and comparative analysis of ARAS and non-ARAS patient groups**.

	ARAS group	Non-ARAS group	*p *value
	(64 cases)	(58 cases)	
Male sex (n [%])	37 (57.8)	23 (39.7)	0.045
Age (years)	72.16 ± 10.91	62.62 ± 13.15	<0.001
Height (cm)	166.17 ± 9.55	166.16 ± 8.59	0.992
Weight (kg)	73.50 (65.00, 82.00)	68.00 (60.00, 75.25)	0.027
BMI (kg/m^2^)	26.44 (24.82, 27.67)	24.56 (23.48, 26.36)	0.002
Curse of hypertension (years)	20.00 (10.00, 30.00)	14.50 (5.50, 20.00)	0.019
Smoke (n [%])	31 (48.4)	23 (39.7)	0.329
Drink (n [%])	13 (20.3)	14 (24.1)	0.611
Diabetes (n [%])	27 (42.2)	22 (37.9)	0.632
CHD (n [%])	26 (40.6)	14 (24.1)	0.053
GLU (mmol/L)	5.93 ± 1.32	5.95 ± 1.50	0.932
HbA1c (%)	7.07 ± 1.18	7.01 ± 1.54	0.098
TG (mmol/L)	1.44 ± 0.89	1.68 ± 2.07	0.386
LDL-C (mmol/L)	2.40 ± 1.04	2.33 ± 0.89	0.692
Cr (µmol/L)	116.34 ± 80.27	70.28 ± 24.16	<0.001
eGFR (kg/m^2^ × body surface area)	65.00 (40.50, 82.25)	88.50 (75.50, 105.00)	<0.001
PRA (pg/mL)	29.00 ± 17.33	13.09 ± 16.20	0.345
PAC (pg/mL)	152.08 ± 82.53	116.38 ± 52.57	0.376
ARR	32.49 ± 7.75	25.11 ± 7.72	<0.001

Abbreviation: ARAS, atherosclerotic renal arterial stenosis; BMI, body mass index; CHD, coronary heart disease; GLU, glucose; HbA1c, glycosylated hemoglobin type A1c; TG, triglyceride; LDL-C, low density lipoprotein cholesterol; Cr, creatinine; eGFR, estimated glomerular filtration rate; PRA, plasma renin activity; PAC, plasma aldosterone concentration; ARR, plasma aldosterone-to-renin ratio levels.

**Fig. 1.**
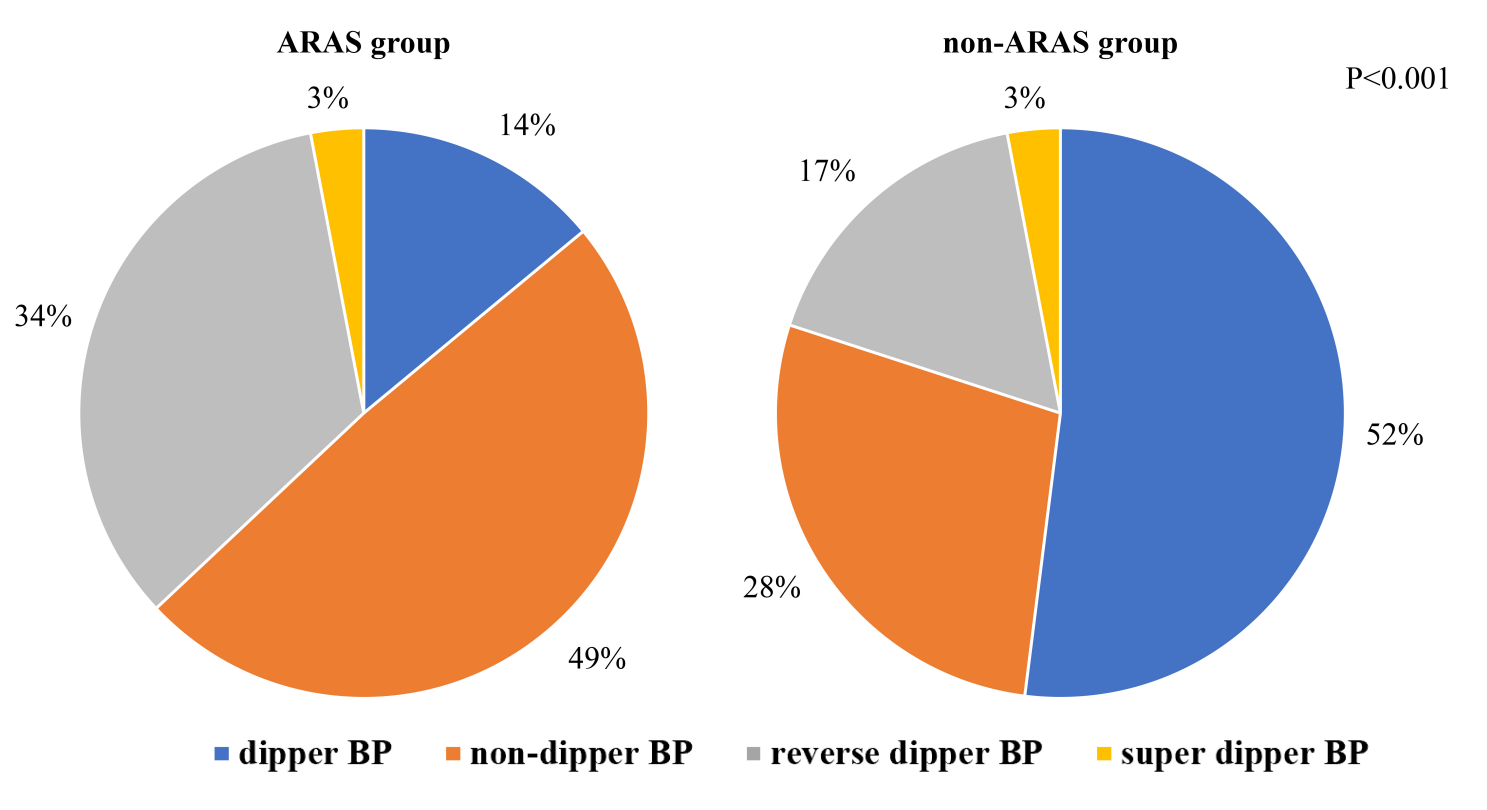
**Prevalence of BPV categories in the ARAS and non-ARAS groups**. This figure illustrates the distribution of blood pressure fluctuation types (dipper and non-dipper) within groups diagnosed with ARAS and non-ARAS. Abbreviation: BP, blood pressure; ARAS, atherosclerotic renal arterial stenosis; non-ARAS, non-atherosclerotic renal artery stenosis; BPV, blood pressure variability.

**Fig. 2.**
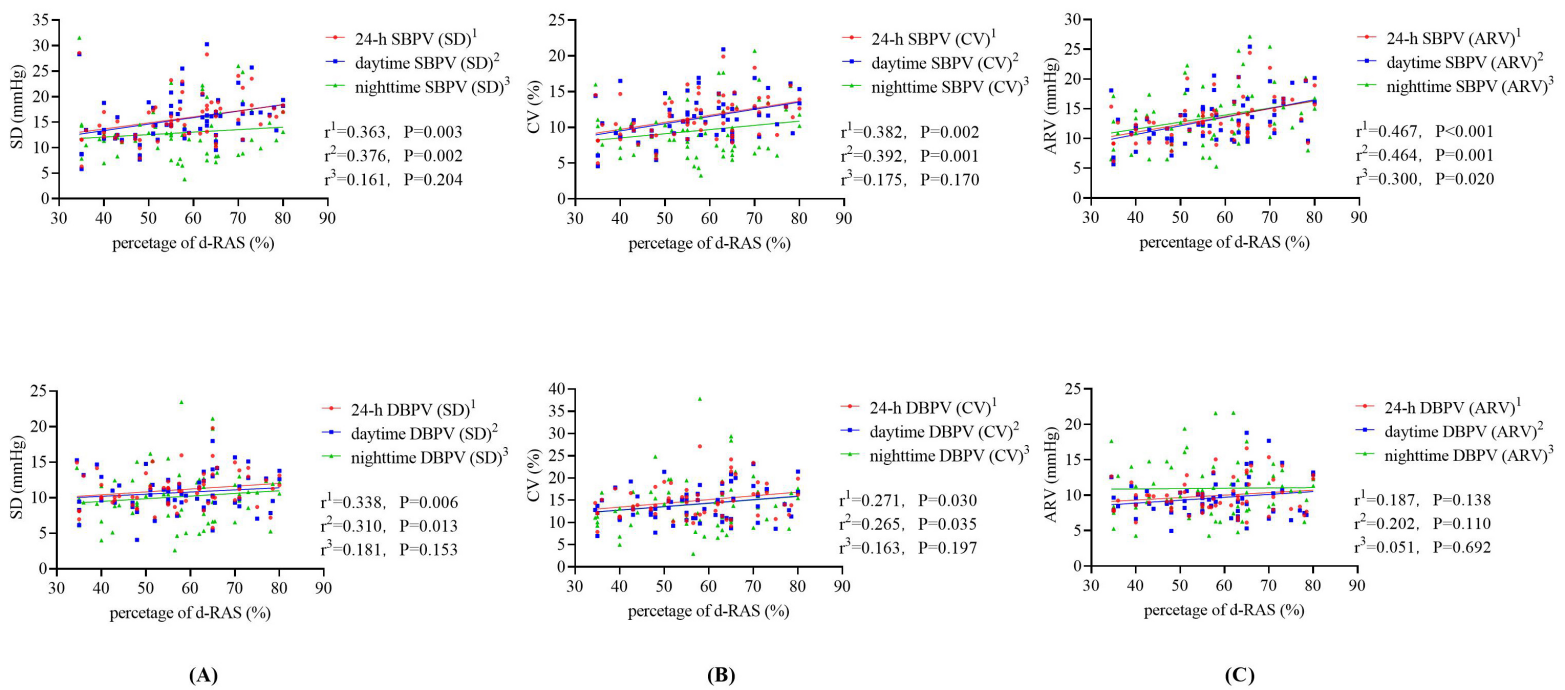
**Scatter plots illustrating the relationship between BPV and d-RAS**. This figure presents scatter plots demonstrating how BPV metrics correlate with d-RAS. (A) SD changes with the d-RAS. (B) CV changes with the d-RAS. (C) ARV changes with the d-RAS. Abbreviation: SBPV, systolic blood pressure variability; DBPV, diastolic blood pressure variability; d-RAS, degree of renal artery stenosis; SD, standard deviation; CV, coefficient of variation; ARV, average real variability; BPV, blood pressure variability..

As shown in Fig. 1, the proportion of dipper hypertension in the ARAS group was significantly lower than that in the non-ARAS group (*p* < 0.001). In the analyses, d-RAS was set as the dependent variable, with SBPV or DBPV as the independent variables. The scatterplot (Fig. 2) showed that the 24-hour (r = 0.363, *p* = 0.003), and daytime (add as appropriate) SBPV was increased with increasing d-RAS. Moreover, the ARV indices that were used to evaluate nighttime SBPV were positively associated with the d-RAS (all *p* < 0.05). Note, all data is represented using SD, CV, and ARV indices.

The correlation between various BPV indices and the degree of d-RAS is detailed in Table 2. The analysis revealed significant positive correlations between unilateral d-RAS and 24-hour SBPV (R^1^ = 0.460, *p* = 0.001; R^1^ = 0.509, *p* < 0.001; R^1^ = 0.677, *p* < 0.001, respectively) and daytime SBPV (R^1^ = 0.512, *p* < 0.001; R^1^ = 0.539, *p* < 0.001; R^1^ = 0.678, *p* < 0.001, respectively). For nighttime SBPV, the ARV indices were significantly associated with unilateral d-RAS (R^1^ = 0.415, *p* = 0.005). The 24-hour DBPV (R^1^ = 0.347, *p* = 0.019; R^1^ = 0.340, *p* = 0.022, respectively), and daytime DBPV, (R^1^ = 0.379, *p* = 0.010; R^1^ = 0.397, *p* = 0.007; R^1^ = 0.319, *p* = 0.033, respectively), were also positively correlated with unilateral d-RAS. However, nighttime DBPV was not associated with unilateral d-RAS (*p* > 0.05). Notably, none of the SBPV or DBPV indices correlated with bilateral d-RAS (*p* > 0.05). Interestingly, the mean nighttime DBP was the only mean BP value that positively correlated with bilateral d-RAS (R^2^ = 0.466, *p* = 0.044), whereas other mean BP measurements showed no significant association with either unilateral or bilateral d-RAS (*p* > 0.05). Note, all data is represented using SD, CV, and ARV indices.

**Table 2.** **Correlation coefficients between BPV indices and the degree of renal artery stenosis in the ARAS group**.

Mean BP and BPV		Degree of renal artery stenosis	
		R (*p*)^1^	R (*p*)^2^
Mean	24-hour SBP	0.023 (0.878)	0.377 (0.111)
	24-hour DBP	0.101 (0.510)	0.451 (0.053)
	Daytime SBP	–0.018 (0.905)	0.352 (0.140)
	Daytime DBP	0.147 (0.335)	0.393 (0.096)
	Nighttime SBP	–0.048 (0.756)	0.351 (0.141)
	Nighttime DBP	0.025 (0.870)	0.466 (0.044)
SD	24-hour SBPV	0.460 (0.001)	0.224 (0.357)
	24-hour DBPV	0.347 (0.019)	0.300 (0.212)
	Daytime SBPV	0.512 (<0.001)	0.169 (0.601)
	Daytime DBPV	0.379 (0.010)	0.008 (0.975)
	Nighttime SBPV	0.184 (0.226)	0.128 (0.601)
	Nighttime DBPV	0.110 (0.473)	0.427 (0.068)
CV	24-hour SBPV	0.509 (<0.001)	0.069 (0.780)
	24-hour DBPV	0.340 (0.022)	–0.019 (0.940)
	Daytime SBPV	0.539 (<0.001)	0.027 (0.914)
	Daytime DBPV	0.397 (0.007)	–0.236 (0.330)
	Nighttime SBPV	0.235 (0.120)	0.005 (0.985)
	Nighttime DBPV	0.139 (0.364)	0.276 (0.253)
ARV	24-hour SBPV	0.677 (<0.001)	0.059 (0.809)
	24-hour DBPV	0.278 (0.065)	–0.183 (0.452)
	Daytime SBPV	0.678 (<0.001)	0.111 (0.652)
	Daytime DBPV	0.319 (0.033)	–0.330 (0.167)
	Nighttime SBPV	0.415 (0.005)	0.071 (0.772)
	Nighttime DBPV	0.016 (0.915)	0.152 (0.534)

This table displays the Pearson correlation coefficients and associated *p*-values illustrating the relationship between BPV indices and the degree of renal artery stenosis. The column labeled R (*p*)^1^ presents data for unilateral renal artery stenosis, while the R (*p*)^2^ column presents data for bilateral renal artery stenosis. Abbreviation: SBP, systolic blood pressure; DBP, diastolic blood pressure; SBPV, systolic blood pressure variability; DBPV, diastolic blood pressure variability; SD, standard deviation; CV, variable coefficient; ARV, average real variability; BPV, blood pressure variability; ARAS, atherosclerotic renal artery stenosis; BP, blood pressure.

The results of the univariate beta regression analysis are shown in **Supplementary Table 2**. Multivariate beta regression analysis was conducted using smoking history, CHD, BMI, plasma aldosterone concentration (PAC), and BPV as independent variables and d-RAS as the dependent variable. As shown in Table 3 shows that smoking history, CHD, and BMI were significant predictors of d-RAS severity in patients with ARAS. After adjusting for other factors, it was found that patients with ARAS who smoked, had higher BMI, or CHD also presented with more severe d-RAS. Similarly, the 24-hour SBPV, assessed through ARV indices (odds ratio [OR] = 1.035, 95% confidence interval [CI]: 1.054–1.607, *p* = 0.035), and daytime SBPV, presented using ARV indices (OR = 1.033, 95% CI: 1.004–1.061, *p* = 0.023), were found to be independent risk factors for d-RAS.

**Table 3.** **Multivariate beta regression analysis of factors influencing the severity of renal artery stenosis in ARAS patients**.

Variations	Estimation of parameter (β)	OR value	95% CI	*p* value
Smoke	0.266	1.305	1.051–1.620	0.016
CHD	0.279	1.322	1.058–1.652	0.014
BMI	0.047	1.048	1.002–1.096	0.040
PAC	0.001	1.001	0.999–1.002	0.344
24-hour SBPV (SD)	0.017	1.017	0.991–1.044	0.191
Smoking history	0.270	1.310	1.054–1.627	0.015
CHD	0.279	1.322	1.004–1.656	0.015
BMI	0.046	1.047	1.001–1.096	0.046
PAC	0.001	1.001	0.999–1.002	0.305
Daytime SBPV (SD)	0.013	1.013	0.989–1.038	0.297
Smoking history	0.259	1.296	1.045–1.606	0.018
CHD	0.275	1.317	1.056–1.641	0.014
BMI	0.040	1.041	0.994–1.089	0.086
PAC	0.001	1.001	0.999–1.002	0.379
24-hour SBPV (CV)	0.035	1.036	0.996–1.076	0.083
Smoking history	0.266	1.305	1.050–1.621	0.016
CHD	0.278	1.320	1.055–1.651	0.015
BMI	0.041	1.042	0.995–1.092	0.084
PAC	0.001	1.001	0.999–1.002	0.317
Daytime SBPV (CV)	0.023	1.023	0.986–1.063	0.225
Smoking history	0.253	1.288	1.041–1.593	0.020
CHD	0.271	1.311	1.055–1.629	0.014
BMI	0.041	1.042	0.996–1.089	0.074
PAC	0.001	1.001	0.999–1.002	0.443
24-hour SBPV (ARV)	0.034	1.035	1.002–1.067	0.035
Smoking history	0.264	1.302	1.054–1.607	0.014
CHD	0.263	1.301	1.048–1.616	0.017
BMI	0.038	1.039	0.993–1.086	0.095
PAC	0.001	1.001	0.999–1.002	0.413
Daytime SBPV (ARV)	0.032	1.033	1.004–1.061	0.023

Multiple beta regression analysis is used for statistics. Abbreviation: CHD, coronary heart diseases; BMI, body mass index; PAC, plasma aldosterone concentration; SBPV, systolic blood pressure variability; SD, standard deviation; CV, variable coefficient; ARV, average real variability; OR, odds ratio; CI, confidence interval; ARAS, atherosclerotic renal artery stenosis.

## 4. Discussion

BPV is recognized as a critical risk factor for cardiovascular disease, and has been independently associated with atherosclerosis, target organ damage, and adverse cardiovascular events, with the risk being independent of the average BP value [23, 24, 25, 26]. A population-based study by Hisamatsu *et al*. [27] found that high variability in family BP values was correlated with carotid artery, aorta, and peripheral atherosclerosis. In our study, both SBPV and DBPV were consistently higher in the ARAS groups across all time points compared to the non-ARAS group. Furthermore, ARAS patients diagnosed using DSA had a significantly higher proportion of reverse dipper BP patterns than those with essential hypertension, indicating greater BPV in ARAS patients, which was consistent with our study [28]. Iwashima *et al*. [29] noted a reduction in BPV in both ARAS and fibromuscular dysplasia patients before and after percutaneous transluminal renal angioplasty. Excessive BPV may be attributed to baroreflex loss, and wide oscillations in BP values promote atherogenic structural and functional vascular wall degeneration [30, 31]. Beyond impaired autonomic control and mean BP, arterial stiffness also serves as a fundamental pathophysiological factor contributing to BPV [32]. In addition, our analysis identified a positive correlation between SBPV at all time points, and both 24-hour and daytime DBPV with unilateral d-RAS.

Interestingly, the average BP reading across all time points was not correlated with unilateral d-RAS. This finding suggests that BPV is a more sensitive and reliable indicator than mean BP in predicting ARAS development. Some authors reported that when the renal blood flow is reduced by over 40%, the renal adaptive response is less effective [33]. In patients with unilateral d-RAS, a significant hemodynamic difference between the narrow and non-narrow sides may suggest the kidney retains compensatory capacity [33]. Conversely, patients with severe bilateral RAS often exhibit poor BP regulation, characterized by consistently high BP values throughout the day and relatively low BPV [7, 8]. This could explain the lack of correlation between bilateral d-RAS and BPV.

There is a significant correlation between BPV and risk factors (relative risk and odds ratio), specifically when BPV was quantified using ARV to assess its association with cardiac events [34]. However, this association was not observed when BPV was expressed as SD [34]. Further supporting this, Mena *et al*. [35] found that SD indices were more sensitive to the recording frequency of ABPM devices and while ARV indices were better predictors of BPV than SD indices.

Several epidemiological studies have shown that the degree of target organ damage is most severe in patients with inverse arytenoid BP values, followed by those with non-arytenoid and arytenoid BP values [36, 37, 38]. However, the effect of super-arytenoid BP on target organ damage and prognosis in patients with hypertension remains controversial. The Wacanda study [39] compared the characteristics of patients with arytenoid, non-arytenoid, reverse arytenoid, and super arytenoid BP values. Interestingly, patients with non-arytenoid and reverse arytenoid BP had more clinical complications and worse prognosis compared to those with arytenoid BP [39]. However, super-arytenoid BP had less multisystem involvement and less damage to the target organs [39]. In this study, the ARAS group contained a higher proportion of patients with non-arytenoid and reverse arytenoid BP compared to the non-ARAS group. However, there were fewer patients with arytenoid BP in the ARAS group than in the non-ARAS group, suggesting a worse prognosis in patients with ARAS. Although only a small number of patients presented with super-arytenoid BP in both groups, further research is needed to elucidate the implications of super-arytenoid BP on renal artery stenosis and its prognosis.

This study found that the ARAS group comprised a higher proportion of older men and with elevated body weight and BMI when compared with the non-ARAS group, indicating these risk factors were associated with ARAS. Aging contributes to atherosclerosis by affecting bone marrow cell development, increasing interleukin-6 levels in blood vessels, and shortening the lifespan of mitochondria [40]. Additionally, the study also found that Cr levels were higher and eGFR levels lower in the ARAS group compared to the non-ARAS group, indicating that ARAS contributes to impaired renal function in patients with hypertension. Dean RH *et al*. [41] conducted a retrospective analysis of 58 patients with impaired renal function, discovering that the rate of decline in eGFR was fivefold higher in patients with RAS compared to those without, suggesting a heightened risk of progressing to end-stage renal disease. While the kidneys possess an intrinsic ability to self-regulate this capacity is compromised when the degree of ARAS reached approximately 60%, leading to evident renal cortical ischemia [42]. The reduction in renal blood flow and resultant tissue hypoxia stimulates renin release, water and sodium retention, and peripheral vascular contraction by activating the renin-angiotensin-aldosterone system (RAAS), leading to ARAS-related hypertension and RAS. A previous study demonstrated that high plasma renin activity could be detected in the narrow lateral renal veins of patients with ARAS, and increased aldosterone levels were associated with reduced renal perfusion in patients with hypertension [43]. Schütten MTJ *et al*. [44] found that a higher ARR is associated with reduced left kidney perfusion. In this study, ARR and its components, including PAC, were higher in patients with ARAS, suggesting that PAC might be a risk factor for d-RAS. The role of the RAAS in RAS development is well-documented, yet the specific contributions of RAAS hormones require further exploration. Additionally, smoking has been identified as a significant risk factor for ARAS. Drummond *et al*. [45] in their study of 931 patients ARAS found that smokers were diagnosed at a younger age than non-smokers and were more likely to suffer from cardiovascular complications or kidney failure, including myocardial infarction, stroke, hospitalization for congestive heart failure, and progressive renal insufficiency. Patients with peripheral atherosclerosis are more prone to coronary heart disease and ARAS [25]. Dzielińska *et al*. [46] found that the incidence of ARAS escalates with increasing number of narrow coronary arteries, with prevalence rates of 10%, 15.8%, and 18.1% among patients with single, double, and triple coronary artery disease, respectively. This study also corroborated the influence of CHD on the progression of ARAS.

This study has some limitations. First, it was a single-center study with a small sample size, and the renal artery CEUS examination could have been influenced by multiple factors, such as subcutaneous fat and abdominal gas. Second, all enrolled patients were hospitalized, and their antihypertensive medication regimen was maintained throughout the study, which might have led to an overestimation of the impact of renal artery stenosis on BPV. Finally, the living conditions of patients during hospitalization were not completely consistent with those outside the hospital, and ABPM could not fully reflect the true BP levels, which may have been influenced by factors like white-coat hypertension. In this study, daytime BP was measured every 30 min, and nighttime BP every 1 h; therefore, the individual differences were not considered while calculating the daytime and nighttime periods. Future research should consider more nuanced approaches to regulating and individualizing blood pressure monitoring to accurately assess BPV.

## 5. Conclusions

This study demonstrated that SBPV and DBPV were positively correlated with d-RAS across various times and indices. A significant positive correlation was observed between unilateral d-RAS and BPV, while no such relationship was evident for bilateral d-RAS. Furthermore, after adjusting for confounding factors, both 24-hour and daytime SBPV, assessed using ARV indices, emerged as independent risk factors for d-RAS. These findings suggest that SBPV and DBPV could serve as novel risk markers for the proactive prevention of ARAS, offering significant public health implications.

## Availability of Data and Materials

The data sets generated and analyzed during the current study are not publicly available because we are doing other research with this dataset but are available from the Wei, Liu and Beijing hospital on reasonable request.

## Supplementary Material

Supplementary material associated with this article can be found, in the online version, at https://doi.org/10.31083/j.rcm2511397.
