# Supplementary material for: Relationship between 24-h Ambulatory Blood Pressure Variability and Degree of Renal Artery Stenosis in Hospitalized Patients with Hypertension
Source: Rev Cardiovasc Med. 2024 Nov 8;25(11):397. doi: 10.31083/j.rcm2511397 (PMC11607514; doi:10.31083/j.rcm2511397)
Supplement: Supplementary file 1 [file 2153-8174-25-11-397-s1.docx]

Supplement table 1. Comparison of the mean BP and BPV between the ARAS and non-ARAS groups

|  | ARAS group | Non-ARAS group | P value |
| --- | --- | --- | --- |
| Mean 24-h SBP (mmHg) | 137.22±16.47 | 128.66±12.92 | 0.002 |
| Mean 24-h DBP (mmHg) | 75.00(67.25, 81.75) | 72.00(66.00, 87.00) | 0.990 |
| Mean daytime SBP (mmHg) | 135.50(125.00, 145.00) | 128.50(119.00, 138.25) | 0.012 |
| Mean daytime DBP (mmHg) | 76.50(70.00, 84.00) | 74.50(67.00, 86.25) | 0.957 |
| Mean nighttime SBP (mmHg) | 135.09±17.82 | 127.02±14.92 | 0.008 |
| Mean nighttime DBP (mmHg) | 72.00(62.25, 79.75) | 71.00(63.75, 84.25) | 0.576 |
| 24-h SBPV(SD) (mmHg) | 15.28(12.46, 17.69) | 9.62(8.29, 10.13) | <0.001 |
| 24-h DBPV(SD) (mmHg) | 11.06(9.28, 12.84) | 7.87(7.11, 8.42) | <0.001 |
| Daytime SBPV(SD) (mmHg) | 14.97(12.48, 18.12) | 9.18(7.71, 8.42) | <0.001 |
| Daytime DBPV(SD) (mmHg) | 10.36(9.28, 12.59) | 7.54(6.79, 8.25) | <0.001 |
| Nighttime SBPV(SD) (mmHg) | 12.90±5.15 | 7.38±2.79 | <0.001 |
| Nighttime DBPV(SD) (mmHg) | 9.59(7.19, 12.30) | 7.13(5.49, 8.16) | <0.001 |
| 24-h SBPV(CV) (%) | 11.04(9.02, 13.48) | 7.45(6.50, 7.87) | <0.001 |
| 24-h DBPV(CV) (%) | 14.39(11.78, 16.70) | 10.50(8.93, 11.55) | <0.001 |
| Daytime SBPV(CV) (%) | 11.04(8.94, 13.14) | 7.10(6.28, 7.69) | <0.001 |
| Daytime DBPV(CV) (%) | 13.68(11.06, 16.62) | 9.95(8.41, 10.60) | <0.001 |
| Nighttime SBPV(CV) (%) | 9.53±3.20 | 5.78±1.94 | <0.001 |
| Nighttime DBPV(CV) (%) | 14.1±5.91 | 9.81±3.20 | <0.001 |
| 24-h SBPV(ARV) (mmHg) | 12.67(11.03, 14.81) | 6.82(6.03, 7.29) | <0.001 |
| 24-h DBPV(ARV) (mmHg) | 9.90±2.39 | 6.73±1.41 | <0.001 |
| Daytime SBPV(ARV) (mmHg) | 12.57(10.06, 15.85) | 6.71(5.83, 7.50) | <0.001 |
| Daytime DBPV(ARV) (mmHg) | 9.57±2.66 | 6.64±1.53 | <0.001 |
| Nighttime SBPV(ARV) (mmHg) | 13.33(10.06, 16.60) | 6.97(5.89, 7.65) | <0.001 |
| Nighttime DBPV(ARV) (mmHg) | 10.66(8.30, 13.39) | 6.91(5.33, 7.75) | <0.001 |

Continuous variables were tested using the t-test and a non-parametric test.

Abbreviation: ARAS, atherosclerotic renal arterial stenosis; SBP, systolic blood pressure; DBP, diastolic blood pressure; SBPV, systolic blood pressure variability; DBPV, diastolic blood pressure variability; SD, standard deviation; CV, coefficient of variation; ARV, average real variability.

Supplement table 2. Univariate Beta regression results of d-RAS and independent variables

| variables | estimate of parameter (β) | OR value | 95%CI | P value |
| --- | --- | --- | --- | --- |
| Female | -0.079 | 0.924 | 0.714–1.180 | 0.534 |
| Age | 0.004 | 1.004 | 0.990–1.018 | 0.464 |
| Course of hypertension | -0.002 | 0.998 | 0.987–1.007 | 0.591 |
| Bilateral d-RAS | -0.022 | 0.978 | 0.767–1.265 | 0.872 |
| BMI | 0.091 | 1.094 | 1.062–1.216 | <0.001 |
| Smoke | 0.446 | 1.562 | 1.251–1.946 | <0.001 |
| Drink | 0.040 | 1.041 | 0.767–1.413 | 0.797 |
| Diabetes | 0.236 | 1.266 | 0.988–1.625 | 0.057 |
| CHD | 0.492 | 1.635 | 1.306–2.057 | <0.001 |
| GLU | 0.031 | 1.031 | 0.393–1.133 | 0.515 |
| HbA1c | 0.021 | 1.021 | 0.920–1.134 | 0.694 |
| TG | 0.031 | 1.031 | 0.897–1.186 | 0.664 |
| LDL-C | -0.095 | 0.909 | 0.809–1.022 | 0.110 |
| Cr | 0.001 | 1.001 | 0.999–1.002 | 0.430 |
| eGFR | -0.003 | 0.997 | 0.993–1.001 | 0.200 |
| PRA | -0.002 | 0.998 | 0.991–1.005 | 0.529 |
| PAC | 0.002 | 1.002 | 1.000–1.003 | 0.035 |
| ARR | 0.003 | 1.003 | 0.987–1.019 | 0.710 |
| Mean 24-h SBP | -0.001 | 0.999 | 0.920–1.007 | 0.887 |
| Mean 24-h DBP | -0.005 | 0.995 | 0.983–1.007 | 0.413 |
| Mean daytime SBP | -0.001 | 0.999 | 0.994–1.005 | 0.790 |
| Mean daytime DBP | -0.002 | 0.998 | 0.986–1.010 | 0.716 |
| Mean nighttime SBP | -0.002 | 0.998 | 0.991–1.005 | 0.585 |
| Mean nighttime DBP | -0.009 | 0.991 | 0.980–1.002 | 0.110 |
| 24-h SBPV(SD) | 0.041 | 1.042 | 1.018–1.093 | 0.003 |
| 24-h DBPV(SD) | 0.038 | 1.039 | 0.986–1.095 | 0.118 |
| Daytime SBPV(SD) | 0.038 | 1.039 | 0.986–1.095 | 0.003 |
| Daytime DBPV(SD) | 0.028 | 1.028 | 0.974–1.084 | 0.246 |
| Nighttime SBPV(SD) | 0.012 | 1.012 | 0.986–1.044 | 0.315 |
| Nighttime DBPV(SD) | 0.015 | 1.015 | 0.988–1.040 | 0.354 |
| 24-h SBPV(CV) | 0.073 | 1.076 | 1.034–1.123 | <0.001 |
| 24-h DBPV(CV) | 0.028 | 1.028 | 1.001–1.059 | 0.084 |
| Daytime SBPV(CV) | 0.064 | 1.066 | 1.025–1.123 | <0.001 |
| Daytime DBPV(CV) | 0.021 | 1.021 | 0.987–1.058 | 0.215 |
| Nighttime SBPV(CV) | 0.028 | 1.029 | 0.987–1.063 | 0.100 |
| Nighttime DBPV(CV) | 0.016 | 1.016 | 0.994–1.032 | 0.142 |
| 24-h SBPV(ARV) | 0.067 | 1.069 | 1.032–1.130 | <0.001 |
| 24-h DBPV(ARV) | 0.037 | 1.038 | 0.994–1.106 | 0.160 |
| Daytime SBPV(ARV) | 0.061 | 1.063 | 1.034–1.117 | <0.001 |
| Daytime DBPV(ARV) | 0.035 | 1.035 | 0.993–1.107 | 0.139 |
| Nighttime SBPV(ARV) | 0.020 | 1.020 | 0.995–1.050 | 0.115 |
| Nighttime DBPV(ARV) | 0.002 | 1.002 | 0.971–1.029 | 0.923 |

The reference value for Female is male. The reference value for bilateral d-RAS is unilateral d-RAS.

Beta regression analysis was used for statistics.

Abbreviation: d-RAS, degree of renal artery stenosis; BMI, body mass index; CHD, coronary heart disease; Cr, creatinine; eGFR, estimated glomerular filtration rate; GLU, glucose; HbA1c, glycosylated hemoglobin type A1c; TG, triglyceride; LDL-C, low density lipoprotein cholesterol; PRA, plasma renin activity; PAC, plasma aldosterone concentration; ARR, plasma aldosterone-to-renin ratio levels; SBP, systolic blood pressure; DBP, diastolic blood pressure; SBPV, systolic blood pressure variability; DBPV, diastolic blood pressure variability; SD, standard deviation; CV, coefficient of variation; ARV, average real variability.
